# Acceptors and Effectors Alter Substrate Inhibition Kinetics of a Plant Glucosyltransferase NbUGT72AY1 and Its Mutants

**DOI:** 10.3390/ijms24119542

**Published:** 2023-05-31

**Authors:** Jieren Liao, Veronika Lederer, Alba Bardhi, Zhiwei Zou, Timothy D. Hoffmann, Guangxin Sun, Chuankui Song, Thomas Hoffmann, Wilfried Schwab

**Affiliations:** 1 Biotechnology of Natural Products, Technische Universität München, Liesel-Beckmann-Str. 1, 85354 Freising, Germany; jieren.liao@tum.de (J.L.); veronika.lederer@tum.de (V.L.); aalbabardhi10@gmail.com (A.B.); ga96loj@mytum.de (Z.Z.); timothy.d.hoffmann@tum.de (T.D.H.); sgx_here@hotmail.com (G.S.); tom.hoffmann@tum.de (T.H.); 2 State Key Laboratory of Tea Plant Biology and Utilization, International Joint Laboratory on Tea Chemistry and Health Effects, Anhui Agricultural University, Hefei 230036, China; sckfriend@163.com

**Keywords:** glycosyltransferase, vanillin, coumarin, sinapaldehyde, fatty acid, effector, substrate inhibition

## Abstract

One of the main obstacles in biocatalysis is the substrate inhibition (SI) of enzymes that play important roles in biosynthesis and metabolic regulation in organisms. The promiscuous glycosyltransferase UGT72AY1 from *Nicotiana benthamiana* is strongly substrate-inhibited by hydroxycoumarins (inhibitory constant *Ki* < 20 µM), but only weakly inhibited when monolignols are glucosylated (*Ki* > 1000 µM). Apocarotenoid effectors reduce the inherent UDP-glucose glucohydrolase activity of the enzyme and attenuate the SI by scopoletin derivatives, which could also be achieved by mutations. Here, we studied the kinetic profiles of different phenols and used the substrate analog vanillin, which has shown atypical Michaelis–Menten kinetics in previous studies, to examine the effects of different ligands and mutations on the SI of NbUGT72AY1. Coumarins had no effect on enzymatic activity, whereas apocarotenoids and fatty acids strongly affected SI kinetics by increasing the inhibition constant *Ki*. Only the F87I mutant and a chimeric version of the enzyme showed weak SI with the substrate vanillin, but all mutants exhibited mild SI when sinapaldehyde was used as an acceptor. In contrast, stearic acid reduced the transferase activity of the mutants to varying degrees. The results not only confirm the multi-substrate functionality of NbUGT72AY1, but also reveal that the enzymatic activity of this protein can be fine-tuned by external metabolites such as apocarotenoids and fatty acids that affect SI. Since these signals are generated during plant cell destruction, NbUGT72AY1 likely plays an important role in plant defense by participating in the production of lignin in the cell wall and providing direct protection through the formation of toxic phytoalexins.

## 1. Introduction

Glycosylation describes a biochemical reaction that strongly alters the physicochemical properties of small molecules, such as water solubility, stability, volatility, bioactivity, and bioavailability, and has proven to be a unique strategy in nature for broadening the chemical spectrum of natural products. This is an important prerequisite for the successful selection of adapted metabolic pathways [1,2]. Several enzyme families have been discovered that can form a variety of glycoside bonds [3,4]. Among others, UDP-dependent glycosyltransferases (UGTs), one of the largest protein families in plants, produce glycosides by transferring a sugar moiety from a donor to an acceptor molecule via an SN2-like mechanism, resulting in an inversion of the configuration of the anomeric carbon [5,6,7,8]. Donors include UDP-glucose, but also UDP-xylose, UDP-glucuronic acid, etc., and acceptors are, e.g., proteins, carbohydrates, lipids and low-molecular metabolites (small molecules) that carry an -OH, -COOH, -SH and -NH_2_ group whereby *O*-, *S*-, *N*-, but also *C*-glycosides and sugar esters, can be formed [4,9]. Enzyme-based glycosylation generally exhibits high stereo- and regioselectivity with both promiscuous UGTs found glycosylating numerous acceptors (generalists) and selective specialists converting few substrates. In general, UGTs show a higher selectivity towards the donor substrate while being more flexible with respect to the acceptor. Although there are several families of glycosyltransferases, the GT1 family in the CAZy classification (www.cazy.org), which includes the UGTs, is of particular interest, because many of its members can glycosylate industrially relevant substances [1,10,11]. UGTs contain a conserved 44-amino-acid-long motif called the PSPG (plant secondary product glycosylation) box, carry a catalytically active His in the N terminus, are inverting Leloir-type glycosyltransferases, and adopt the GT-B fold [11]. Notably, UGTs are involved in the biosynthesis of a number of plant metabolites, such as flavonoids, alkaloids, terpenoids, and polyphenols. Thus, UGTs promote plant growth and development by modifying, detoxifying, transporting, and storing secondary metabolites and volatiles, and by protecting against biotic and abiotic stresses [12,13].

Since the advent of genome sequencing, and due to advances in sequencing techniques, the number of putative UGTs has increased exponentially, but only a few of them have been studied in depth. The characterization of UGTs could reveal a wealth of enzymes that could be harnessed for industrial purposes. Recently, interest in UGTs has focused mainly on their applications as catalysts in the biotechnological production of physiologically active metabolites such as steviosides, cardiotonic steroids, and *C*-glycosides [14,15,16]. Important criteria in these studies were substrate tolerance, regioselectivity, and enzyme reaction mechanisms. In similar studies, we discovered the promiscuous UGT72AY1 from *Nicotiana benthamiana*, which is thought to be involved in lignin biosynthesis, as shown in its homologs from *Arabidopsis thaliana* [17,18]. The glycosylation of monolignols roughly followed a Michaelis–Menten (MM)-like scheme, whereas strong substrate inhibition (SI) was observed when hydroxycoumarins such as scopoletin were used as acceptors (inhibitory constant *K_i_* < 20 µM) [19]. A detailed biochemical analysis of this unusual enzyme by hydrogen/deuterium exchange mass spectrometry (HDX-MS) and mutational analyses revealed the strong UDP-glucose glucohydrolase activity of the enzyme in the absence of an acceptor substrate, which could be attenuated by apocarotenoid effector molecules [20]. However, the same effectors increased the enzymatic activity of the protein toward hydroxycoumarins by increasing the inhibitory constant *K_I_* and thus reducing SI. A similar effect was observed in an F87I and a chimeric mutant (chimera A; Figure S1) containing a sequence segment of a homologous enzyme that showed only weak SI. Based on HDX-MS analyses and in silico modelling that identify amino acids interacting with the substrate, mutants N27D, R91F, R91M, and R91A were generated. The amino acids, N27 and R91, are thought to play a role in SI [19].

Since 20% of enzymes are inhibited by their own substrates, SI appears to have important biological functions and probably represents a biologically relevant regulatory mechanism [21]. However, since the molecular causes of this inhibition have been poorly investigated, we have performed further studies to find additional substrates of NbUGT72AY1 that show SI, and to clarify whether vanillin, which is structurally related to scopoletin, behaves similarly to hydroxycoumarin when glucosylated by this enzyme. The aim of the study was to investigate the effects of coumarins and various mutants on the enzymatic activity of UGT72AY1 toward vanillin and sinapaldehyde and to analyze how apocarotenoid and fatty acid effector molecules alter the kinetics of the reaction. The results show that NbUGT72AY1 is a flexible protein whose catalytic properties are modified by substrate and effector molecules, allowing it to sense the environment and adapt the enzyme activity accordingly.

## 2. Results

### 2.1. The Substituents of the Phenolic Substrates Dictate the Enzyme Kinetics of NbUGT72AY1

**Figure 1:**
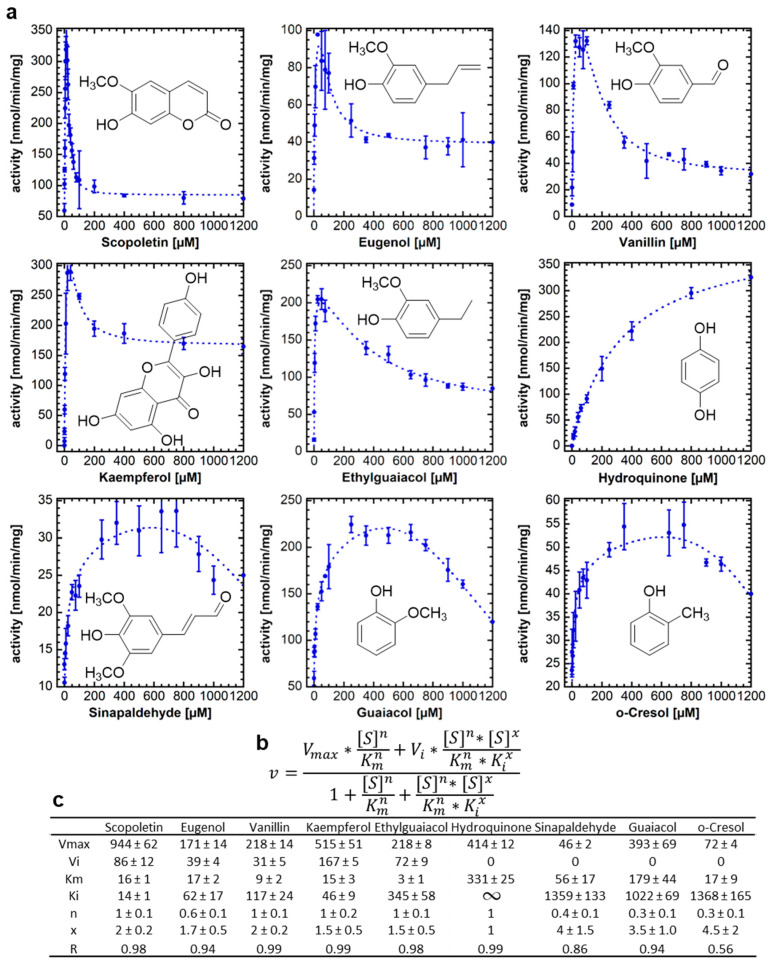
**Enzymatic activity of NbUGT72AY1 toward various phenolic substrates.** NbUGT72AY1 was incubated with increasing concentrations of substrates and the product UDP was determined by UDP-Glo^TM^ assay. (**a**) Plots of acceptor substrate concentration versus reaction rate. (**b**) Equation used for fitting the data (partial uncompetitive inhibition model with Hill coefficients) [23]. (**c**) Kinetic data obtained by fitting the data to the equation shown in (**b**).

In a previous study, we showed that the promiscuous NbUGT72AY1 can glucosylate phenolics as well as short-chain alcohol, terpenoids, and apocarotenoids [17]. Since NbUGT72AY1 was strongly substrate-inhibited by hydroxycoumarins, vanillin, and carvacrol, but only weakly by monolignols [19], we performed further biochemical studies to reveal structure–function relationships. Enzyme activity studies carried out by UDP-Glo^TM^ glucosyltransferase assay, with naturally occurring substrates structurally related to scopoletin and vanillin, showed a wide range of different activity profiles, ranging from Michaelis–Menten (MM) kinetics (hydroquinones) over weakly inhibited (sinapaldehyde, guaiacol, and o-cresol) to strongly substrate-inhibited profiles (scopoletin, eugenol, and vanillin) (Figure 1a). The equation that best explained the data for all substrates combines the two-binding site kinetic model for sequential ordered binding [22] and the Hill equation [23]. The equation contains two Hill coefficients, n and x, where x accounts for the possibility that substrate binding can also be cooperative in the inhibitory mode (Figure 1b). The equation becomes an MM equation when n and x are equal to 1, *V_i_* is equal to 0, and *K_i_* approaches infinity, as in the case of hydroquinone (Figure 1c). Complete substrate inhibition can be recognized by the fact that *V_i_* is equal to 0 (sinapaldehyde, guaiacol, and o-cresol). All ortho-substituted phenols show SI kinetics, while para-substituted hydroquinone is not inhibited by the substrate. Kaempferol is a special case, as two products, kaempferol-3-*O*-glucoside and -7-*O*-glucoside, were formed (Figure S2). The replacement of the methoxy group in guaiacol with a methyl group, as in o-cresol, led to a strong decrease in *V_max_* (393 ± 69 vs. 72 ± 4 nmol/min/mg) and *K_m_* (179 ± 44 vs. 17 ± 9 µM), while other parameters were not significantly different, highlighting the importance of an ortho-substituent for substrate binding in the catalytic site. A third substituent appears to enhance the SI, as shown by the comparisons of guaiacol with ethylguaiacol/eugenol/vanillin. A fourth substituent, as in scopoletin and sinapaldehyde, leads to different outcomes.

### 2.2. Coumarins Have No Effect on UDP-Glucose Glucosyltransferase Activity and Substrate Inhibition of NbUGT72AY1

**Figure 2:**
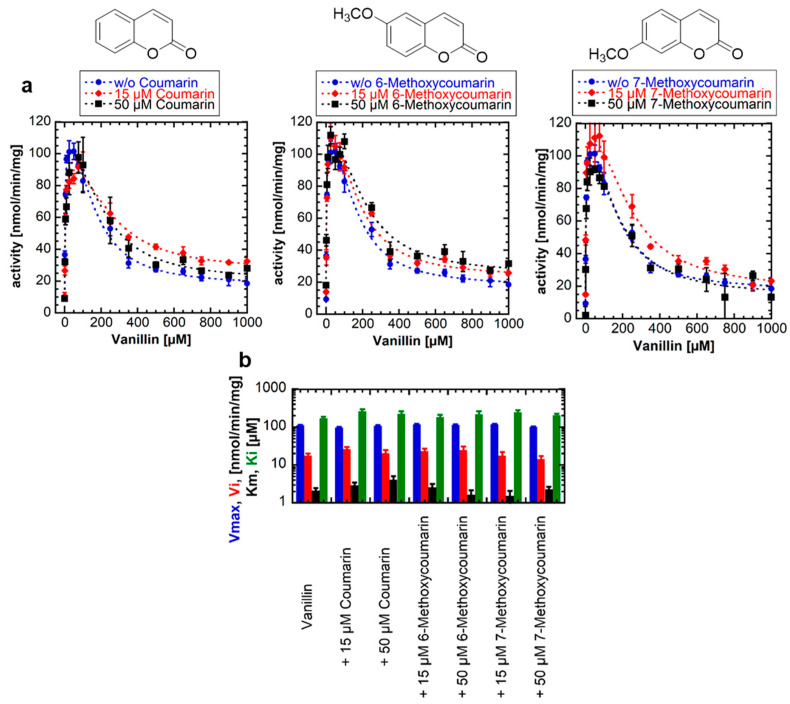
**Enzyme activity of NbUGT72AY1 toward vanillin in the presence of different coumarins.** (**a**) NbUGT72AY1 was used to glucosylate vanillin in the presence of different concentrations of coumarin, 6-methoxycoumarin, and 7-methoxycoumarin. Enzyme activity was determined by UDP-Glo^TM^ Glycosyltransferase assay and fitted to the equation shown in Figure 1. (**b**) Kinetic parameters of NbUGT72AY1 using vanillin as acceptor substrate in the presence of coumarins. Please note that the y-axis is displayed logarithmically to display the entire range of values. The colors of the bars are explained on the y-axis. Experimental values were fitted to the equation shown in Figure 1 (n = 1; x = 2). Parameters are not significantly different (*p* < 0.01).

One possible hypothesis for the strong SI by scopoletin is based on a second (allosteric) substrate binding site on the NbUGT72AY1 protein [19]. To uncouple the catalytic and inhibitory modes of substrate binding, we used vanillin as the substrate and coumarins as possible inhibitors. However, enzyme activity assays performed by UDP-Glo^TM^ in the absence and presence of coumarin, 6-methoxycoumarin, and 7-methoxycoumarin showed that the kinetic parameters of UGT catalysis were not affected by the coumarins (Figure 2). Using a one-tailed *t*-test (*p* < 0.01), the *V_max_*, *V_i_*, *K_m_*, and *K_i_* values of the samples with coumarins were not significantly different from those of the samples without coumarins. Thus, we concluded that either there is no second binding site on NbUGT72AY1, or the coumarins are unable to bind to the allosteric site. Since the number and position of substituents on the phenol ring are essential for binding to the allosteric site of the enzyme, as shown in the previous section for hydroquinone, it is likely that coumarins, unlike hydroxycoumarins, do not interact with NbUGT72AY1.

### 2.3. Mutant NbUGT72AY1 Proteins Show Different Enzyme Kinetics with Vanillin but Similar Enzyme Reaction Curves with Sinapaldehyde

**Figure 3:**
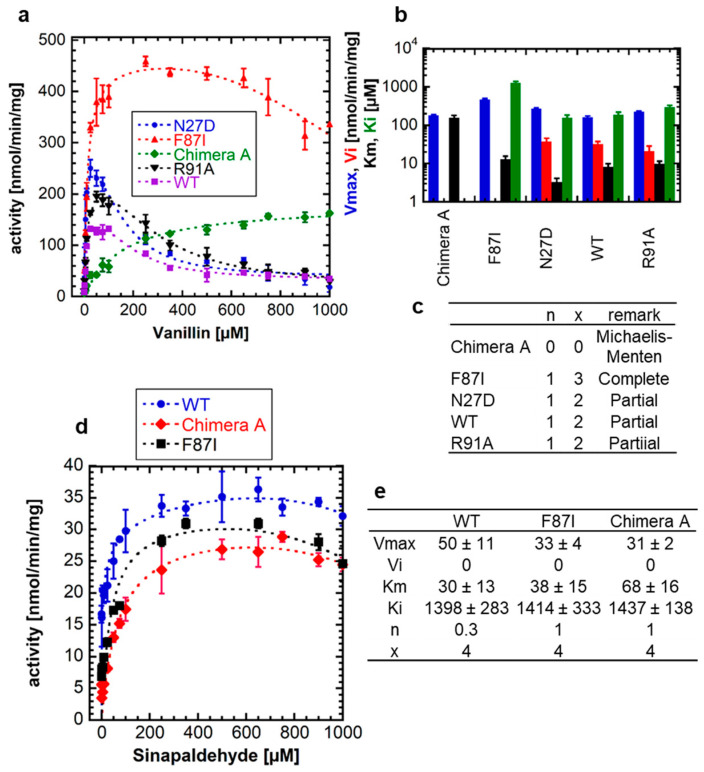
**Enzyme activity of wild-type NbUGT72AY1 and selected mutants toward vanillin and sinapaldehyde.** (**a**) Enzyme activity versus substrate (vanillin) concentration plot for wild-type (WT) NbUGT72AY1 and selected mutants. The N27D and R91A mutant show a similar substrate-inhibited enzyme kinetics as the WT protein. (**b**) Kinetic parameters of NbUGT72A1 and mutant enzymes using vanillin as acceptor substrate. Please note that the y-axis is displayed logarithmically to show the entire range of values. The colors of the bars are explained on the y-axis. (**c**) Values were fitted to the equation shown in Figure 1. Mutants show Michaelis–Menten, complete (V_i_ = 0) and partial uncompetitive substrate (V_i_ > 0) inhibition kinetics. (**d**) Enzyme activity versus substrate (sinapaldehyde) concentration plot for WT NbUGT72AY1 and selected mutants. Experimental data were determined by UDP-Glo^TM^ Glycosyltransferase assay. (**e**) Kinetic parameters of NbUGT72A1 and mutant enzymes using sinapaldehyde as acceptor substrate. Values were fitted to the equation shown in Figure 1. Parameters are not significantly different (*p* < 0.01).

In a previous study, an F87I and a chimera A mutant of NbUGT72AY1 showed reduced SI with scopoletin compared with the wild-type (WT) enzyme [19]. In the F87I mutant, an essential amino acid in the active site was exchanged, whereas the chimera A mutant contained a sequence part of StUGT72AY2 from *Solanum tuberosum*, which is a homolog of NbUGT72AY1 but has only a weak SI. Therefore, in a new experiment, we investigated whether the mutants behave similarly when the natural substrate analog vanillin is used (Figure 3a). 

While mutants N27D and R91A showed a strong SI similar to the WT with comparable kinetic data, except for the *V_max_* of N27D, which was significantly different from the value of the WT, F87I and chimera A showed a weak SI (*K_i_* = 1261 ± 128 µM) and MM kinetics, respectively (Figure 3b,c). Thus, the catalytic activity of NbUGT72AY1 and its mutants toward vanillin resembles that of the enzymes toward scopoletin, implying that the phenolic aldehyde should interact with the same amino acids of the proteins as the phenolic lactone. In contrast, WT NbUGT72AY1, as well as the F87I and chimera A mutants showed mild SI with sinapaldehyde (Figure 3d). 

Although structurally related, monolignol is thus likely to bind more weakly to the putative allosteric site than scopoletin, since the *K_i_* values are significantly higher (1398 ± 283 µM, 1414 ± 333 µM, and 1437 ± 138 µM for WT, F87I, and chimera A, respectively) (Figure 3e) than those for scopoletin to the WT variant (16 ± 1 µM) (Figure 1a,c).

### 2.4. Apocarotenoids and Fatty Acids Enhance the Glucosyltransferase Activity and Reduce Substrate Inhibition of NbUGT72AY1 with Vanillin 

**Figure 4:**
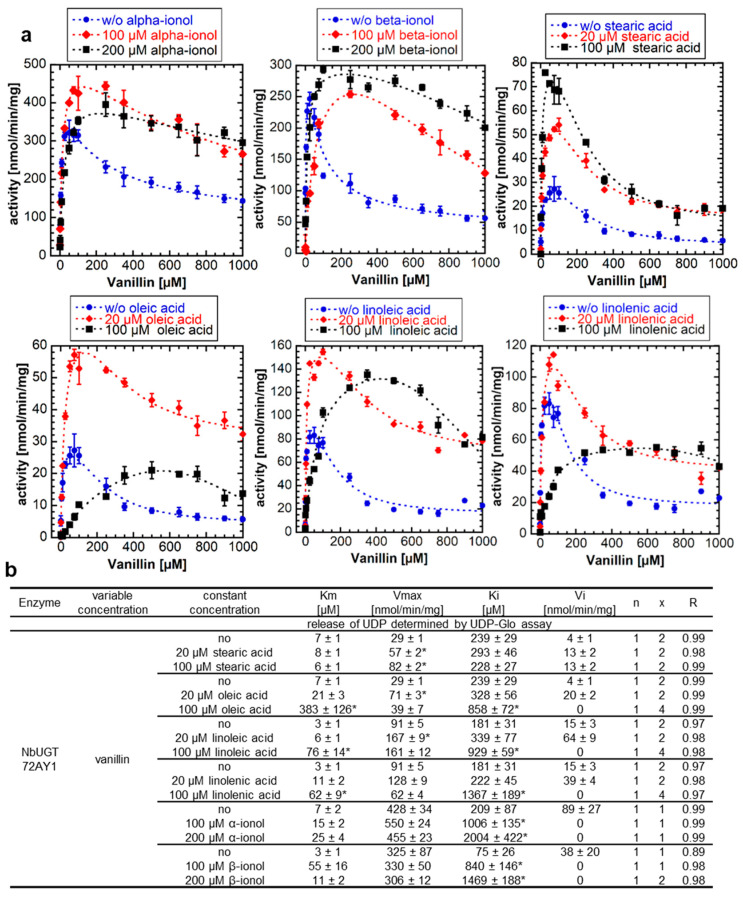
**Enzyme activity of NbUGT72AY1 toward vanillin in the presence of apocarotenoids and fatty acids.** (**a**) NbUGT72AY1 was used to glucosylate vanillin in the presence of α- and β-ionol, stearic acid, oleic acid, linoleic acid, and linolenic acid. Experimental data were determined by UDP-Glo^TM^ Glycosyltransferase assay and fitted to the partial uncompetitive inhibition model shown in Figure 1. (**b**) Kinetic parameters of NbUGT72AY1 using vanillin as substrate with various effectors. Asterisks (*) indicate that the values are statistically significantly different according to a *t*-test (*p* < 0.01) from the values for the samples without addition of the effectors.

Apocarotenoids, including α- and β-ionol, have been shown to increase the glucosylation activity of NbUGT72AY1 toward scopoletin by decreasing SI due to increasing *K_i_* levels [20] and UDP-glucuronosyltransferase 1A1 activity is inhibited by fatty acids [24]. Therefore, we analyzed the effect of the naturally occurring effectors α- and β-ionol (100 µM and 200 µM each) and fatty acids such as stearic acid, oleic acid, linoleic acid, and linolenic acid (20 µM and 100 µM each) on the enzymatic activity of NbUGT72AY1 toward vanillin (Figure 4a). The addition of α- and β-ionol to the reaction resulted in a concentration-dependent increase in *K_i_* and reduced *V_i_* to zero (complete uncompetitive SI) (Figure 4b). Similarly, the addition of C18 fatty acids altered the course of the enzymatic reaction curve, but in different ways. 

Saturated stearic acid promoted the glycosylation activity of NbUGT72AY1 with vanillin as *V_max_* increased in a concentration-dependent manner (Figure 4b). The unsaturated fatty acids also resulted in a statistically significant increase in *V_max_* upon addition of 20 µM, but also in a concentration-dependent increase in *K_i_* that was statistically significant at 100 µM. Moreover, the reaction curve changed to a complete uncompetitive SI upon addition of 100 µM of the unsaturated fatty acids. Thus, it appears that, in the case of oleic, linoleic, and linolenic acid, more than one cause contributes to the altered response curve. The increased catalytic activity of NbUGT72AY1, noted after addition of stearic and oleic acid, was independently confirmed by LC-MS analysis. The reduced product formation after excessive addition of oleic acid (100 µM) was also corroborated by LC-MS analysis (Figure S3).

### 2.5. The Enzymatic Activity of NbUGT72AY1 Mutants Is Either Unaffected or Reduced by Fatty Acids

**Figure 5:**
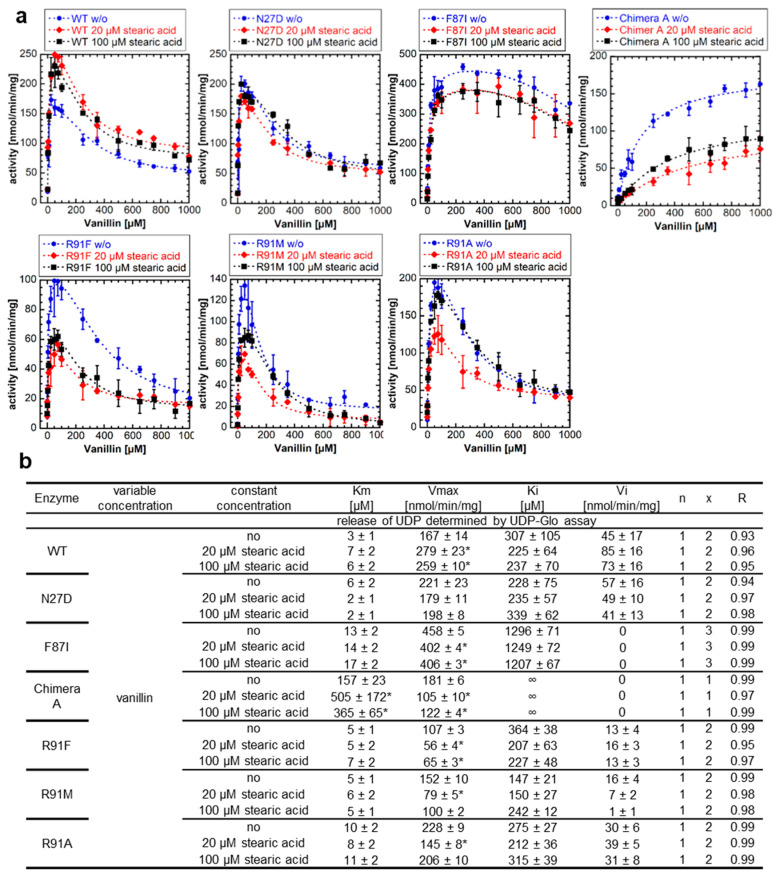
**Enzyme activity of NbUGT72AY1 mutants toward vanillin in the presence of putative effectors.** (**a**) Wild-type (WT) and mutant proteins were used to glucosylate vanillin in the presence of stearic acid. Experimental data were determined by UDP-Glo^TM^ Glycosyltransferase assay and fitted to the equation shown in Figure 1. (**b**) Kinetic parameters of WT and mutant proteins using vanillin as substrate with stearic acid. Asterisks (*) indicate that the values are statistically significantly different according to a *t*-test (*p* < 0.01) from the values for the samples without addition of the effectors.

Since fatty acids such as stearic acid altered the enzymatic activity of WT NbUGT72AY1 by decreasing SI in the previous experiment, we next tested the effects on NbUGT72AY1 mutants (F87I and chimera A) that exhibit reduced SI (Figure 5a). The results of the enzyme activity assays confirmed the promoting effect of stearic acid for the NbUGT72AY1 WT, as explained by the significantly increased *V_max_* values, and showed that the saturated acid did not affect the catalytic activity of the N27D mutant, but did alter the activities of the F87I and chimera A mutants (Figure 5b). 

The *V_max_* values were significantly lowered after the addition of stearic acid in the F87I mutant, which has only a weak SI, whereas the *V_max_* and *K_m_* values were considerably reduced and increased, respectively, in the chimera A mutant. In the R91 mutants (R91F, R91M, and R91A), in which an amino acid presumably important for SI had been replaced, the addition of stearic acid had no effect or decreased activity, with the *V_max_* values significantly reduced at 20 µM addition. Thus, it can be concluded that stearic acid has a positive, promoting effect on the catalytic activity of the WT NbUGT72AY1 enzyme, which exhibits strong SI but has no effect, or an inhibitory effect, on mutants in which amino acids putatively involved in SI have been mutated.

## 3. Discussion

**Figure 6:**
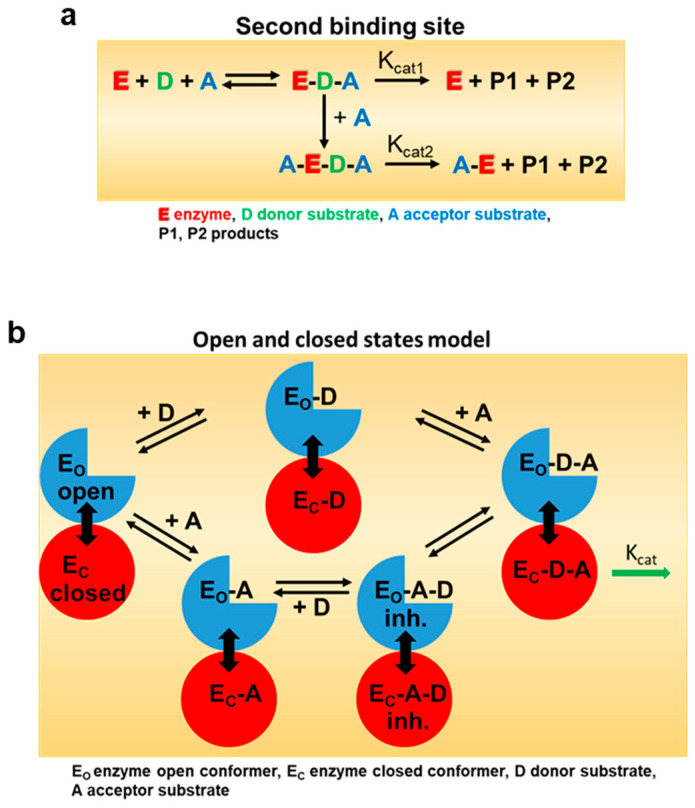
**Model for enzymatic substrate inhibition.** (**a**) In the classical model for substrate inhibition the enzyme has catalytic activity when one substrate is bound, but reduced (K_cat2_ < K_cat1_) or even no activity K_cat2_ = 0) if two are bound (adapted from [22]). The color code is explained in the sub figure. (**b**) In the open and closed states model, inhibitory concentrations of acceptor substrate A lead to a faster and more cooperative domain closure by donor substrate D, leading, in turn, to an increased population of the closed inhibited state (E_C_-A-D). Too rapid a premature closure of the domain could interfere with substrate-binding mechanisms (adapted from [27]).

Promiscuous enzymes are of particular interest for the biotechnological production of chemicals, agrochemicals, and pharmaceuticals, as they catalyze reactions in a stereoselective manner and can be used for the manufacture of a wide range of industrially relevant products due to their substrate tolerance. However, a significant number of biocatalysts are inhibited by their substrates at high concentrations, limiting their potential applications. Since the molecular mechanisms of SI are not fully understood, the targeted elimination of this limiting enzyme property by rational design is difficult. The best-known example of SI is the inhibition of phosphofructokinase by ATP, which leads to the suppression of glycolysis and, thus, to the cessation of ATP production [25]. The most commonly cited model of SI is the two binding site model with a catalytic and allosteric site [22]. The binding of the substrate to the allosteric binding site in the enzyme (E) or in the enzyme–substrate complex (ES) forms an inhibitory complex in which the catalyzed reaction is either very slow or completely suppressed (Figure 6). In alternative models, excess substrate molecules interact with enzyme forms other than the enzyme–substrate complex, such as the reaction intermediate (EI) or the enzyme–product complex (EP). All these models have in common a second substrate molecule that is bound to the enzyme. The inhibitory effects of substrates are attributed to the accumulation of a catalytically incompetent combination of enzyme, cofactor, and substrate. Such inappropriate termination complex formation has been reported for multi-substrate and multi-product enzymes with multiple binding sites [26]. However, a recent model based on conformational motions of proteins has shown that an allosteric site is not essential for SI. By using single-molecule FRET spectroscopy, it has been demonstrated that acceptor substrates can facilitate the domain closure of a kinase at lower concentrations of the donor substrate, which can affect the proper substrate-binding mechanics required for the reaction [27]. NbUGT72AY1 lends itself here as a model to better understand SI as it shows extreme SI (Figure 1).

### 3.1. NbUGT72AY1 Exhibits Michaelis–Menten and Substrate Inhibition Kinetics Depending on Substrate Structure

NbUGT72AY1 is a promiscuous enzyme that glucosylates phenols as well as short-chain alcohols and terpenes [17]. Studies on enzyme activity showed that MM and SI kinetics were obtained depending on the substrate structure (Figure 1). While only the para-substituted hydroquinone showed a hyperbolic curve, ortho- and tri-substituted phenols exhibited both weak and strong SI. Hydroquinone thus presumably binds exclusively in the catalytic center, or an excess of benzene-1,4-diol has no effect on the dynamics of domain closure. The measured *K_m_* values for NbUGT72AY1 substrates are relatively low (<200 µM), with the exception of hydroquinone, suggesting that this enzyme has a high affinity for a number of ortho-substituted phenols. UGT72 enzymes are thought to be involved in the modification of flavonoids and the lignin formation by glucosylation of monolignols [28,29], as shown for homologous enzymes from *Arabidopsis thaliana* [30,31]. Similarly, NbUGT72AY1 glucosylates flavonoids and monolignols such as kaempferol and sinapaldehyde, albeit with different efficiencies and kinetics [19] (Figure 1). However, NbUGT72AY1 may also be implicated in the detoxification of the airborne phenols produced by forest fires, as guaiacol and ethylguaiacol are metabolized efficiently and the enzyme is constitutively expressed in the stem of the tobacco plant. The glucosylation of airborne volatiles after uptake by plants has been demonstrated in the leaves of grapevine, tomato and tea plants [32,33,34].

The strong SI of NbUGT72AY1 for scopoletin was related to its putative function in plant defense [13,20]. Thus, the neighboring cells of tissues damaged by an herbivore might protect themselves by glucosylation of the phytoalexin synthesized in response to the attack. However, if the damage is too severe, it might be more beneficial to build a physical barrier of dead cells, which could explain the significantly reduced activity at high scopoletin concentrations [35].

### 3.2. Blocking the Inhibitory Action of Substrates

Since both scopoletin and vanillin exhibit SI, we attempted to uncouple the inhibitory effect of vanillin from the catalytic activity by adding different coumarin derivatives to the reaction solution. However, the enzyme activity curves and kinetic parameters were not significantly different in the presence of the coumarins (Figure 2). The coumarins are either unable to replace vanillin at the allosteric site or the second binding site is not existent. In the case of the second hypothesis for SI, this would mean that coumarins have no influence on the dynamics of enzyme movement. However, mutants of NbUGT72AY1 generated to suppress SI in the WT enzyme toward scopoletin (F87I and chimera A) [19] also showed reduced SI or no inhibition at all with vanillin (Figure 3). However, the mutants did not exhibit altered kinetics of sinapaldehyde glucosylation compared with WT. In the N27D and R91A mutants, the amino acids putatively involved in SI had been exchanged [19], but only in N27D was *V_max_* significantly increased compared with WT. Amino acid F87 is part of the active site and was identified by HDX-MS analysis [19], whereas chimera A contains a segment of a homologous enzyme that showed only weak SI [19]. Based on in silico analyses, it was hypothesized that NbUGT72AY1 has an allosteric site that shares F91 and amino acids of the sequence inserted into chimera A with the catalytic center [19]. The hypothesis is supported by the observation that the crystal structure of a human sulfotransferase (SULT1A1) contains two substrate molecules and the residue Phe-247 of SULT1A1, which interacts with both p-nitrophenol molecules, is important for substrate inhibition [26]. The results obtained for vanillin confirm the data determined for scopoletin and show that SI can be reduced or even abolished by the replacement of individual amino acids. This was also shown for tyrosine hydroxylase [36], betaine aldehyde dehydrogenase [37], salutaridine reductase [38], human sulfotransferase [26], D-3-phosphoglycerate dehydrogenase [39], lactate dehydrogenase [40], and haloalkane dehalogenase [41].

### 3.3. Effectors Increase the Enzymatic Activity of Substrate-Inhibited NbUGT72AY1 but Decrease the Catalytic Activity of Mutants That Exhibit Attenuated Substrate Inhibition

Recently, we showed that the SI of scopoletin in NbUGT72AY1 was decreased by apocarotenoids, which could be explained by an increase in the inhibitory constant *K_i_* [20]. In terms of the two-substrate binding site model, this implies that binding to the allosteric site is restricted by apocarotenoids. In the case of the second model, this means that the effectors prevent the early closure of the catalytic center when the acceptor substrate is present in excess. In this study, we demonstrated that a decrease in SI and an increase in enzymatic activity is also possible through the addition of fatty acids (Figure 4). Thus, 20 µM of stearic, oleic, linoleic, and linolenic acids increased *V_max_*. However, at 100 µM of unsaturated fatty acids, *K_m_* and *K_i_* increased significantly and *V_i_* decreased to zero compared to the sample without effectors. An exception was stearic acid, where a concentration-dependent increase in enzyme activity was observed. A possible explanation for the different behavior is the micelle and vesicle formation of the long-chain fatty acids (Figure S4). Saturated and unsaturated fatty acids form self-assembling structures such as micelles, vesicles and oil droplets at different pH values of the medium above their critical micelle concentrations (CMC), and critical vesicle concentration (CVC), depending on the concentration of the acids, the temperature, and the ionic strength of the buffer [42,43] (Figure S4). Therefore, different CVCs and CMCs for fatty acids are found in the literature, but it seems that CMCs increase with the number of double bonds of the fatty acids and pH (Table S1). At concentrations below the CMC (20 µM), when the acids are dissolved as single molecules, they can readily interact with NbUGT72AY1 and promote the activity of the enzyme (Figure 4). At concentrations above the CMC (100 µM), when micelles and vesicles have formed, the diffusion from the micelles and vesicles, respectively, leads to an obvious increase in *K_m_* and *K_i_* values in the case of the unsaturated acids (Figure 4 and Figure S4). Stearic acid enhances glucosylation activity even at a concentration of 100 µM. However, stearic acid does not promote activity in NbUGT72AY1 mutants in which the amino acids thought to play a role in SI have been mutated, but actually decreases *V_max_* in the case of the F87I and chimera A mutant and increases *K_m_* for chimera A (Figure 5). Thus, this is an un-competitive inhibition of the chimera A mutant (binding to the enzyme–substrate complex only), which exhibits MM kinetics and a non-competitive inhibition of F87I (binding to the enzyme and enzyme–substrate complex). The inhibition of enzyme activities by free fatty acids has long been known [44] and, recently, lipoxygenases were shown to be regulated by fatty acids through interaction with an N-terminal binding domain [45]. Furthermore, stearic acid suppressed the enzymatic activity of a thioesterase when a C-terminal lipid binding domain was absent, suggesting that this hydrophobic domain abolished the inhibitory effect of stearic acid [46]. In the future, HDX-MS will clarify whether the segment replaced in chimera A is a possible binding site for fatty acids.

## 4. Materials and Methods

### 4.1. Cloning and Protein Expression of UGT72AY1 

Cloning of NbUGT72AY1 from *Nicotiana benthamina* (accession MT945401) with vector pGEX-4T-1, and the protein expression in *Escherichia coli BL21(DE3) pLysS*, were performed according to [17]. Mutants (N27D, F87I, chimera A, R91A, R91F, and R91M) were generated according to [17,19] (Figure S5).

### 4.2. Enzyme assays by UDP Glo^TM^ Glycosyltransferase Assay

The kinetics of UGT72AY1 with vanillin and effectors were measured using the UDP-Glo^TM^ Glycosyltransferase Assay (Promega, Mannheim, Germany). The optimal enzymatic reaction conditions were determined as described [17]. The 100 µL assay contained 50 mM Tris pH 7.5, 0.5 µg protein, a defined concentration of effector, and different concentrations of vanillin and 100 mM UDP-glucose, which was added to start the incubation at 40 °C and 500 rpm for 10 min. Finally, the reaction was stopped by 12.5 µL 0.6 M HCl and an addition of 12.5 μL 1 M Trizma base pH 10.7 was used to adjust the pH. UDP detection reagent (UDR) was used to quantify the released UDP during the catalysis in a 384-well plate (384-Well Plates, Corning 4513, Sigma–Aldrich, Taufkirchen, Germany) and incubated for 30 min in the dark before the luminescence signal was measured by the CLARIOstar plate reader (BMG Labtech, Ortenberg, Germany). Kinetic data were calculated with KaleidaGraph (https://www.synergy.com/; accessed 22 May 2023; v4.5.4).

### 4.3. Liquid Chromatography-Mass Spectrometry Analysis

After centrifugation (20 min at 5000× *g*), the samples prepared for the UDP-Glo assay were used for LC-MS analysis according to [17]. Vanillin, sinapaldehyde, and kaempferol glucosides were identified according to [19] (Figure S2).

### 4.4. Enzyme Kinetics Analysis

A two-site model (Figure 1) was used to explain the substrate inhibition phenomenon of NbUGT72AY1 [19]. Here, [S] is the concentration of the varied substrate, *V_max_* is the maximal reaction rate, and *K_m_* represents the substrate concentration at which the reaction rate is ½ *V_max_*. The parameter *V_i_* is the reaction velocity in the presence of inhibition, *K_i_* is the inhibition constant which is the inhibitor concentration required to decrease the maximal rate of the reaction to ½ of the uninhibited value. The equation presumes the sequential binding of substrate molecules, i.e., the inhibitory site cannot be occupied until the reaction site is filled. By adding cooperativity-describing Hill coefficients, an equation was obtained that best described the measured data. The superscript n is a Hill coefficient, and x is another Hill coefficient that allows for the possibility that the binding of substrate in the inhibitory mode may also be cooperative [47]. To obtain convergence for the equation in Figure 1, the value of x was fixed, which was determined empirically to give a best fit (lowest variance). The kinetic parameters were determined under optimum conditions and were calculated with KaleidoGraph version 4.5.4 from Synergy Software (Eden Prairie, MN, USA). The data were derived from at least three repeats. Statistical analysis was performed using Student’s *t*-test (*p* < 0.01).

$$v=\frac{V_{max}\;*\;\frac{{\left[S\right]}^{n}}{K_{m}^{n}}+V_{i}\;*\;\frac{{\left[S\right]}^{n}\;*\;{\left[S\right]}^{x}}{K_{m}^{n}\;*\;K_{i}^{x}}}{1+\frac{{\left[S\right]}^{n}}{K_{m}^{n}}+\frac{{\left[S\right]}^{n}\;*\;{\left[S\right]}^{x}}{K_{m}^{n}\;*\;K_{i}^{x}}}$$

## 5. Conclusions

Overall, the results show that vanillin behaves similarly to scopoletin as a substrate for NbUGT72AY1. These substrates show SI with the WT, which is reduced by apocarotenoids, is only weakly observed in the F87I mutant, and is not seen at all in the chimera A mutant. Coumarins do not affect enzymatic activity, but this does not completely rule out a second binding site. Fatty acids, on the other hand, promote catalytic activity depending on free fatty acid molecules, which is why aggregations of fatty acids above their CMC lead to altered reaction rates. Since the enzyme exhibits SI with a range of substrates, this opens up unique opportunities for the regulation of the enzyme by effectors that can attenuate inhibition. NbUGT72AY1 is a multi-substrate enzyme whose enzymatic activity can be fine-tuned by external, naturally occurring metabolites such as apocarotenoids and fatty acids that affect SI. These signals are generated upon plant cell destruction, which is why NbUGT72AY1 likely plays an important role in plant defense as it may be involved in the production of lignin in the cell wall and may provide direct protection through the formation of toxic phytoalexins.

## Data Availability

Data are contained within the article or Supplementary Material.

## Supplementary Materials

The following supporting information can be downloaded at: https://www.mdpi.com/article/10.3390/ijms24119542/s1. References [48,49,50,51,52] are cited in the supplementary materials.
