# Supplementary material for: Acceptors and Effectors Alter Substrate Inhibition Kinetics of a Plant Glucosyltransferase NbUGT72AY1 and Its Mutants
Source: Int J Mol Sci. 2023 May 31;24(11):9542. doi: 10.3390/ijms24119542 (PMC10253357; doi:10.3390/ijms24119542)
Supplement: Supplementary file 1 [file ijms-24-09542-s001.zip › Supplementary Material Figures S1-S5.pdf]

SUPPLEMENTAL MATERIAL

Acceptors and Effectors Alter Substrate Inhibition Kinetics of a Plant Glucosyltransferase NbUGT72AY1 and Its Mutants

Jieren Liao [1], Veronika Lederer [1], Alba Bardhi [1], Zhiwei Zou [1], Timothy D. Hoffmann [1], Guangxin Sun [1,†], Chuankui Song [2], Thomas Hoffmann [1] and Wilfried Schwab [1,*]

[1] Biotechnology of Natural Products, Technische Universität München, Liesel-Beckmann-Str. 1, 85354 Freising, Germany; jieren.liao@tum.de (J.L.); veronika.lederer@tum.de (V.L.); aalbabardhi10@gmail.com (A.B.); ga96loj@mytum.de (Z.Z.); timothy.d.hoffmann@tum.de (T.D.H.); sgx_here@hotmail.com (G.S.); tom.hoffmann@tum.de (T.H.)

[2] State Key Laboratory of Tea Plant Biology and Utilization, International Joint Laboratory on Tea Chemistry and Health Effects, Anhui Agricultural University, Hefei 230036, China; sckfriend@163.com

* Correspondence: wilfried.schwab@tum.de

† Current address: Department of Molecular Biophysics and Biochemistry, Yale University, New Haven, CT 06510, USA.

Figure S1. Schemes of the generated mutants.

Figure S2. LC-MS analysis of substrates and products of NbUGT72AY1.

Figure S3. Enzyme activity of NbUGT72AY1 toward vanillin in the presence of fatty acids as determined by LC-MS analysis.

Figure S4. Enzymatic reaction in the presence of micelles and vesicles formed by fatty acid effectors.

Figure S5: Verification of NbUGT72AY1 mutant protein by SDS-PAGE.

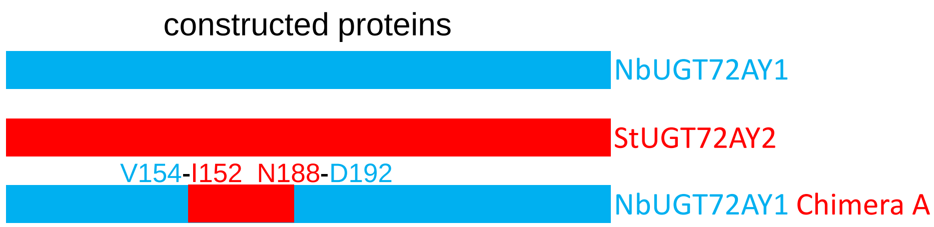

Figure S1. Schemes of the generated mutants. Chimera A contains a part of StUGT72AY2. StUGT72AY2 is a homolog of NbUGT72AY1 but shows no substrate inhibition with scopoletin [19]

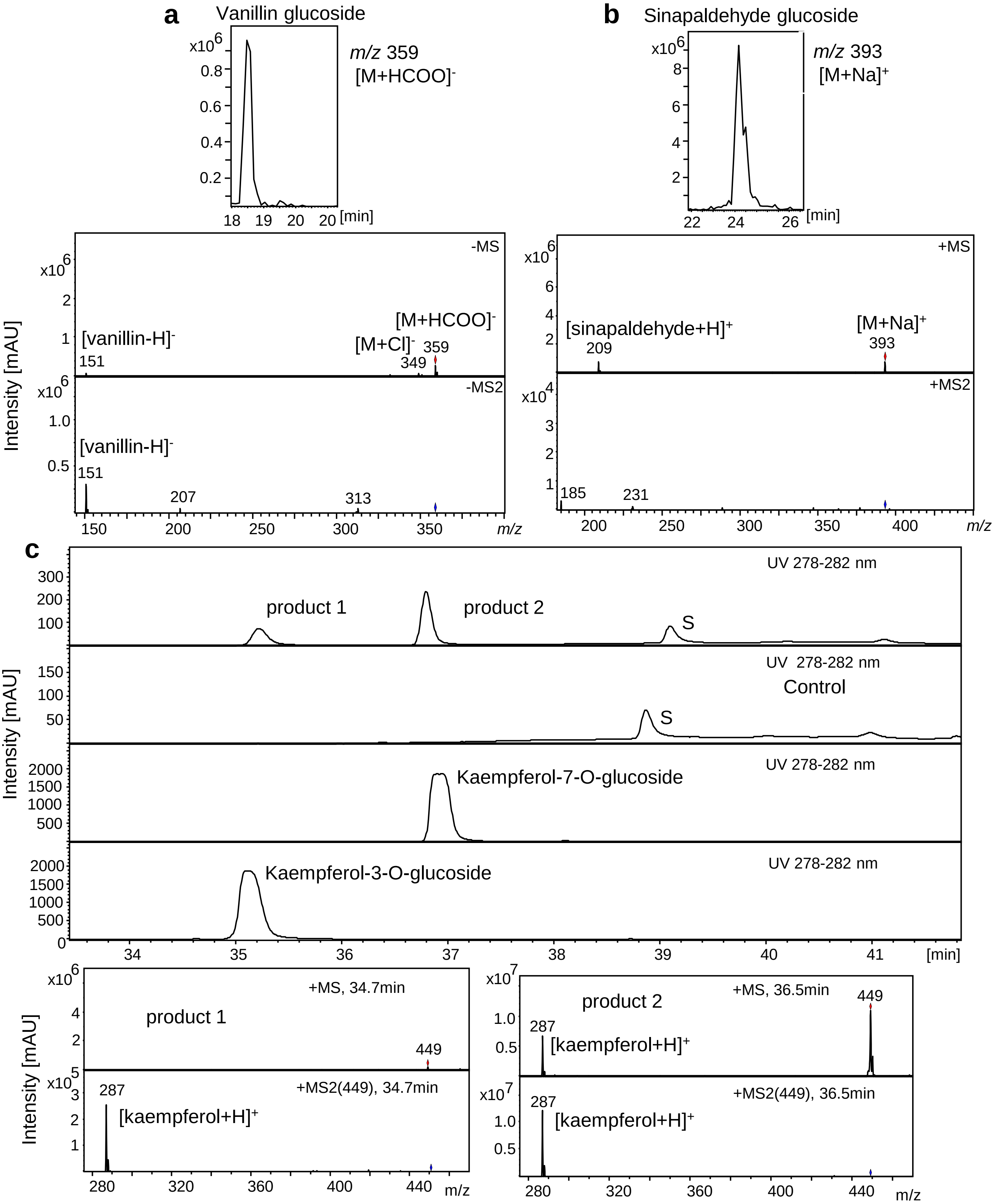

Figure S2. LC-MS analysis of substrates and products of NbUGT72AY1. Chromatogram (top), mass spectrum (MS) and product ion spectra (MS2) acquired in the positive (+) and negative mode (-). (**a**) Vanillin glucoside and (**b**) sinapaldehyde glucoside. (**c**) Ultraviolet detection (UV) at 278-282 nm of kaempferol (S) and two products. The products were identified as product 1 kaempferol-3-O-glucoside and product 2 kaempferol-7-O-glucoside by comparison with chromatographic data obtained for the reference compounds.

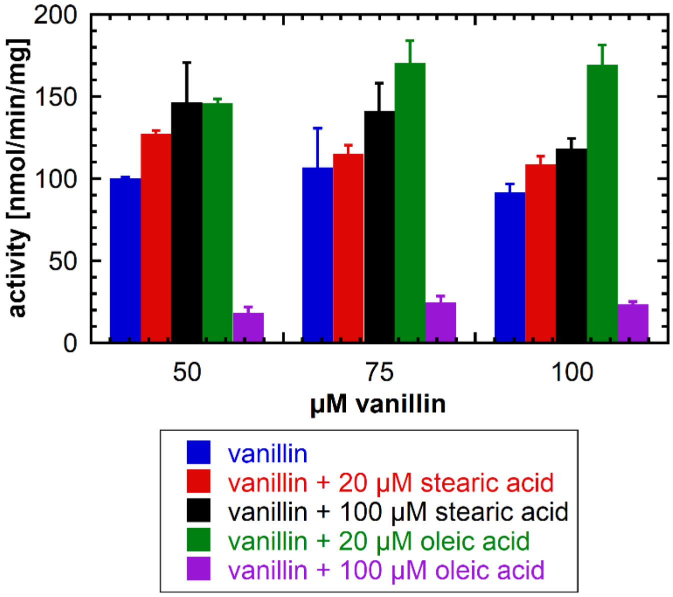

Figure S3. Enzyme activity of NbUGT72AY1 toward vanillin in the presence of fatty acids as determined by LC-MS analysis. NbUGT72AY1 was used to glucosylate vanillin in the presence of stearic acid and oleic acid. Experimental data were determined by LC-MS analysis.

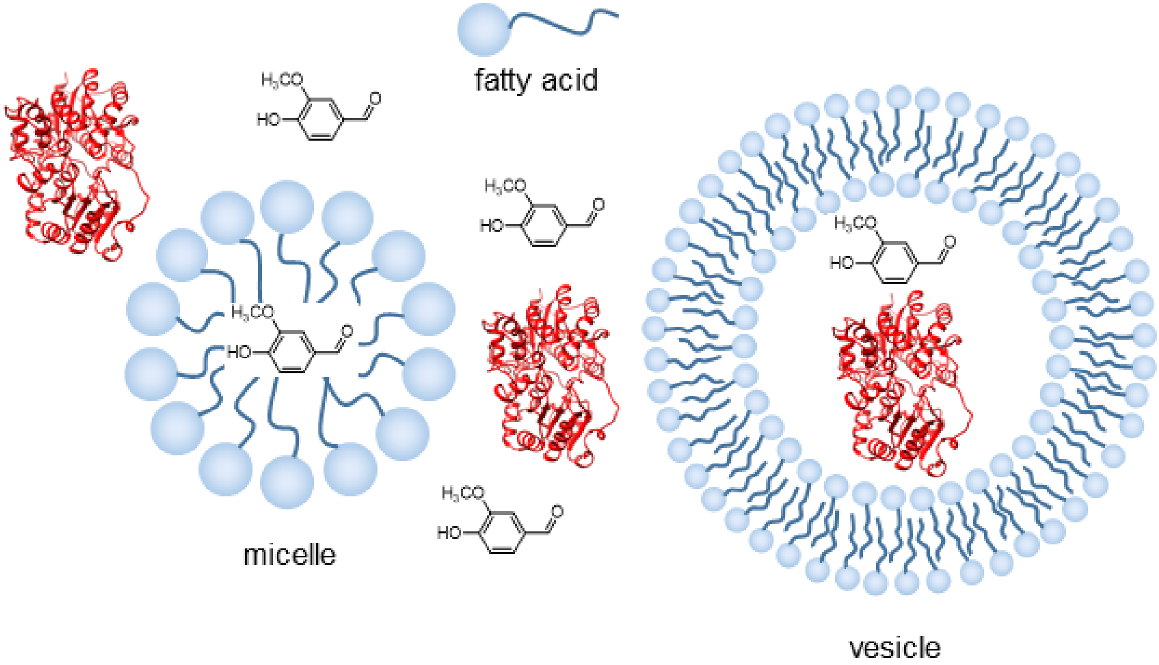

Figure S4. Enzymatic reaction in the presence of micelles and vesicles formed by fatty acid effectors.

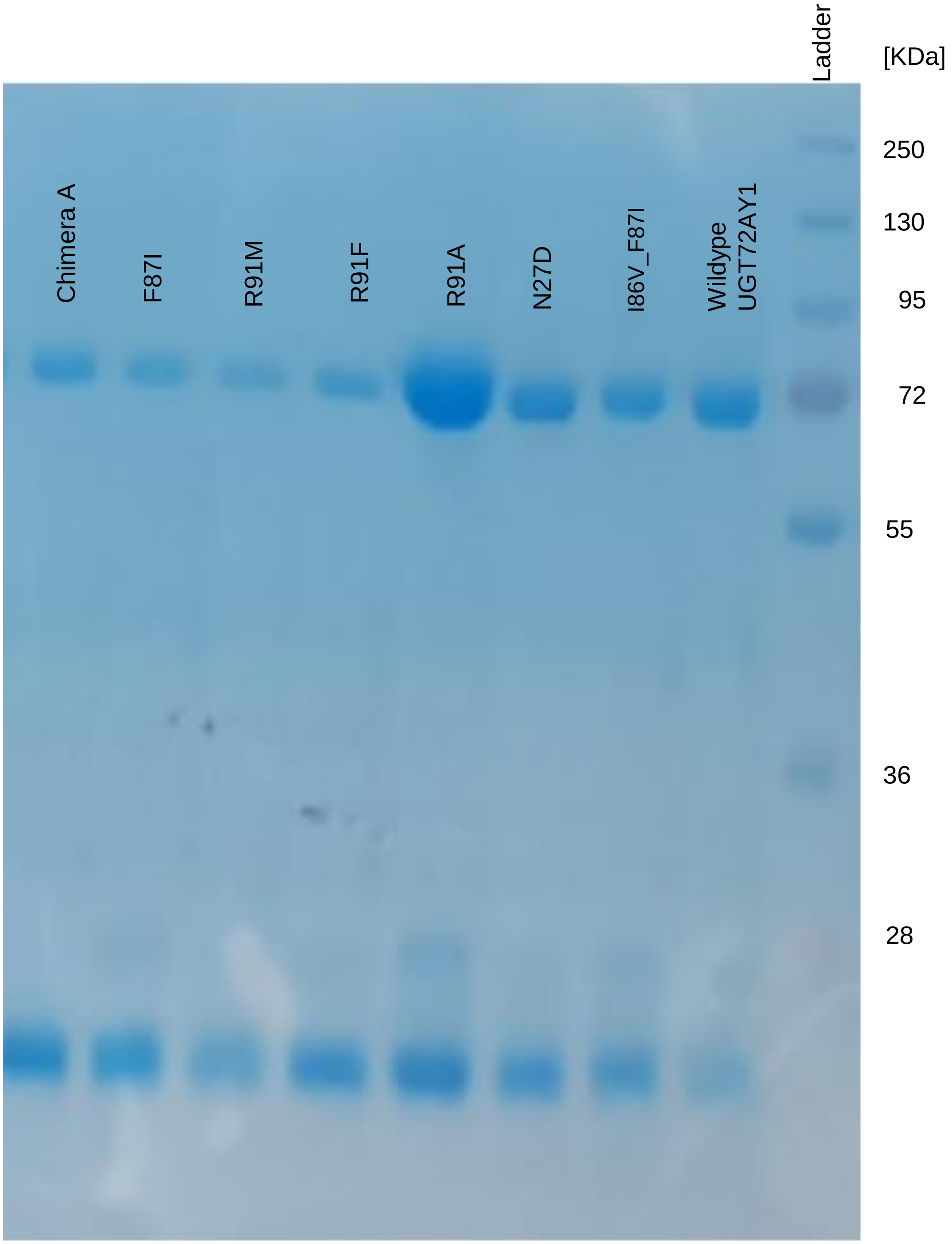

Figure S5: Verification of NbUGT72AY1 mutant protein by SDS-PAGE. The gel shows the result of the SDS-PAGE that was obtained after GST-affinity purification of the recombinant proteins. Ladder, PageRuler Plus Prestained Protein Ladder
