# Supplementary material for: Acceptors and Effectors Alter Substrate Inhibition Kinetics of a Plant Glucosyltransferase NbUGT72AY1 and Its Mutants
Source: Int J Mol Sci. 2023 May 31;24(11):9542. doi: 10.3390/ijms24119542 (PMC10253357; doi:10.3390/ijms24119542)
Supplement: Supplementary file 1 [file ijms-24-09542-s001.zip › Supplementary Material Table S1.pdf]

SUPPLEMENTAL MATERIAL

Acceptors and Effectors Alter Substrate Inhibition Kinetics of a Plant Glucosyltransferase NbUGT72AY1 and Its Mutants

Jieren Liao 1, Veronika Lederer 1, Alba Bardhi 1, Zhiwei Zou 1, Timothy D. Hoffmann 1, Guangxin Sun 1,†, Chuankui Song 2, Thomas Hoffmann 1 and Wilfried Schwab 1,*

1 Biotechnology of Natural Products, Technische Universität München, Liesel-Beckmann-Str. 1,
85354 Freising, Germany; jieren.liao@tum.de (J.L.); veronika.lederer@tum.de (V.L.);
aalbabardhi10@gmail.com (A.B.); ga96loj@mytum.de (Z.Z.); timothy.d.hoffmann@tum.de (T.D.H.);
sgx_here@hotmail.com (G.S.); tom.hoffmann@tum.de (T.H.)
2 State Key Laboratory of Tea Plant Biology and Utilization, International Joint Laboratory on Tea Chemistry and Health Effects, Anhui Agricultural University, Hefei 230036, China; sckfriend@163.com
* Correspondence: wilfried.schwab@tum.de
† Current address: Department of Molecular Biophysics and Biochemistry, Yale University, New Haven, CT 06510, USA.

Table S1. Critical vesicle concentration (CVC) and critical micelle concentration (CMC) of long-chain fatty acids.

Table S1. Critical vesicle concentration (CVC) and critical micelle concentration (CMC) of long-chain fatty acids. Values in mM.

	[1]	[2]						[3]			[4]	[5]
	CVC pH 9	CMC pH7	CMC pH8	CMC pH 9	CMC pH10	CMC pH11	CMC pH12	CMC pH8	CMC pH9	CMC pH10	CMC pH ns	CMC pH7.6
stearic acid											0.5	
oleic acid	0.13	0.0471	0.045	0.052	0.057	0.074	0.115					0.086
linoleic acid	0.51							0.021	0.060	0.117		0.093
linolenic acid	1.23											0.150

ns not specified

References

1. Teo, Y.Y.; Misran, M.; Low, K.H.; Zain, S.M. Effect of Unsaturation on the Stability of C 18 Polyunsaturated Fatty Acids Vesicles Suspension in Aqueous Solution. Bulletin of the Korean Chemical Society 2011, 32, 59–64, doi:10.5012/bkcs.2011.32.1.59.
2. Theander, K.; Pugh, R.J. The Influence of pH and Temperature on the Equilibrium and Dynamic Surface Tension of Aqueous Solutions of Sodium Oleate. J. Colloid Interface Sci. 2001, 239, 209–216, doi:10.1006/jcis.2000.7543.
3. Verhagen, J.; Vliegenthart, J.F.; Boldingh, J. Micelle and acid-soap formation of linoleic acid and 13-L-hydroperoxylinoleic acid being substrates of lipoxygenase-1. Chem. Phys. Lipids 1978, 22, 255–259, doi:10.1016/0009-3084(78)90014-2.
4. Fameau, A.-L.; Ventureira, J.; Novales, B.; Douliez, J.-P. Foaming and emulsifying properties of fatty acids neutralized by tetrabutylammonium hydroxide. Colloids and Surfaces A: Physicochemical and Engineering Aspects 2012, 403, 87–95, doi:10.1016/j.colsurfa.2012.03.059.
5. Glick, J.; Santoyo, G.; Casey, P.J. Arachidonate and related unsaturated fatty acids selectively inactivate the guanine nucleotide-binding regulatory protein, Gz. J. Biol. Chem. 1996, 271, 2949–2954, doi:10.1074/jbc.271.6.2949.
